# MACSIMS : multiple alignment of complete sequences information management system

**DOI:** 10.1186/1471-2105-7-318

**Published:** 2006-06-23

**Authors:** Julie D Thompson, Arnaud Muller, Andrew Waterhouse, Jim Procter, Geoffrey J Barton, Frédéric Plewniak, Olivier Poch

**Affiliations:** 1 Laboratoire de Biologie et Genomique Structurales, Institut de Génétique et de Biologie Moléculaire et Cellulaire, Illkirch, France; 2 The Laboratory of Molecular Biology, Genetic Analysis & Modelling, Luxembourg; 3 Post Genomics & Molecular Interactions Centre, School of Life Sciences, University of Dundee, UK

## Abstract

**Background:**

In the post-genomic era, systems-level studies are being performed that seek to explain complex biological systems by integrating diverse resources from fields such as genomics, proteomics or transcriptomics. New information management systems are now needed for the collection, validation and analysis of the vast amount of heterogeneous data available. Multiple alignments of complete sequences provide an ideal environment for the integration of this information in the context of the protein family.

**Results:**

MACSIMS is a multiple alignment-based information management program that combines the advantages of both knowledge-based and *ab initio *sequence analysis methods. Structural and functional information is retrieved automatically from the public databases. In the multiple alignment, homologous regions are identified and the retrieved data is evaluated and propagated from known to unknown sequences with these reliable regions. In a large-scale evaluation, the specificity of the propagated sequence features is estimated to be >99%, i.e. very few false positive predictions are made. MACSIMS is then used to characterise mutations in a test set of 100 proteins that are known to be involved in human genetic diseases. The number of sequence features associated with these proteins was increased by 60%, compared to the features available in the public databases. An XML format output file allows automatic parsing of the MACSIM results, while a graphical display using the JalView program allows manual analysis.

**Conclusion:**

MACSIMS is a new information management system that incorporates detailed analyses of protein families at the structural, functional and evolutionary levels. MACSIMS thus provides a unique environment that facilitates knowledge extraction and the presentation of the most pertinent information to the biologist. A web server and the source code are available at .

## Background

Systems biology is an emerging discipline whose goal is to integrate different levels of information in order to gain a deeper understanding of how biological systems function [1]. By studying the various parts of a biological system (e.g., gene and protein networks, metabolic pathways, organelles, cells or organisms) and the relationships and interactions between them, it is hoped that eventually an understandable model of the whole system can be developed. Such systems-level studies have been made possible thanks to the new information resources that are being created from the raw data produced by different high throughput technologies in fields such as transcriptomics, proteomics, or interactomics. Effective analyses in systems biology require the computational power to analyze these comprehensive and massive datasets, and the capacity to integrate heterogeneous data into a usable knowledge format [2]. To address this problem, information management systems are now being developed in a number of areas for the integration of biological data resources with analytical tools using computational, bioinformatic and mathematical methods and the presentation of the results in an intuitive, user-friendly format for the biologist. For example, MARS (microarray analysis, retrieval, and storage system) [3] provides a comprehensive suite for storing, retrieving, and analyzing microarray data. MOLE (mining, organizing, and logging experiments) [4] has been developed to help protein scientists manage the large amounts of laboratory data being generated due to the acceleration in proteome research. GIMS (Genome Information Management System) [5] is an object database that integrates genomic data for *Saccharomyces cerevisiae *with data on the transcriptome, protein-protein interactions, metabolic pathways and annotations, such as gene ontology terms and identifiers. GeneNotes [6] allows users to collect and manage diverse biological information about genes/ESTs. AutoFACT [7] combines information from various databases and assigns functional definitions to gene sequences.

In the context of the new systems-level biology, more detailed analyses are now needed that describe gene function at different levels, such as specific residue interactions, the biochemical function, the role of the gene product in complexes and pathways and the implications for the development and activities of the organism. To achieve this, such diverse information as 3D structures, protein interactions and modifications, or mutations and their associated phenotypes must be assembled, classified and made available to the biologist. Multiple alignments of molecular sequences represent an ideal basis for the reliable integration of all this information [8]. By placing the sequence in the framework of the overall family and by analysing the variation/conservation at different positions, multiple alignments can be used to identify important structural or functional motifs that have been conserved through evolution, and to highlight particular non-conserved features resulting from specific events or perturbations. For example, in protein secondary structure prediction, the use of aligned sequences allows better application of the propensities of particular residues for particular secondary structures and improved identification of patterns of hydrophobicity [9]. Furthermore, the reliability of numerous *ab initio *predictions has been improved by calculation of a consensus prediction over all the sequences in a multiple alignment, e.g. for the characterization of membrane proteins [10], sub-cellular localization [11] or post-transcriptional modifications [12].

Recent advances in multiple sequence alignment methods [e.g. [13-16]] have led to significant improvements in alignment efficiency and accuracy, and it is now possible to construct rapid, reliable alignments of large sets of complete sequences. These new multiple alignments provide the basis for most state-of-the-art systems for protein characterization [e.g. [17-22]]. Thanks to the increasing adoption of common data standards and exchange formats, a number of systems have also been described recently that allow the integration and visualization of other information in the context of a family of proteins e.g. Pfaat [23], the Bio-dictionary [24], MyHits [25]or SRS3D [26].

We have developed MACSIMS, a new multiple alignment-based information management system that combines knowledge-based methods with complementary *ab initio *sequence based predictions for protein family analysis. MACSIMS takes advantage of the recently developed Multiple Alignment Ontology (MAO) [27], to integrate different types of data in the framework of the multiple alignment. A wide range of information, from taxonomic data and functional descriptions to individual sequence features, such as structural domains and active site residues, is mined from the public databases using the SRS Sequence Retrieval System [28]. A number of new algorithms have been developed for reliable data validation, consensus predictions and rational propagation of information from the known to the unknown sequences. The goal of the present paper is to demonstrate the accuracy and reliability of these algorithms, and to introduce some potential applications of this powerful information management system.

The information collected from the public databases contains not only high quality, experimentally validated data, but also less reliable data from high-throughput experimental technologies and computational predictions. In MACSIMS, the mined data is first validated by comparing the information retrieved for each sequence. At the same time, any local alignment errors are detected and the well-aligned homologous regions in the alignment are identified using the LEON algorithm [29]. The consistent information is then propagated from the known to the unknown proteins only within these safe regions. MACSIMS thus provides a reliable environment for a rational transfer of information, which is robust to errors in the input data and to alignment errors. The reliability of the MACSIMS data management system is demonstrated using a large-scale test set of 83 automatic alignments based on the BAliBASE [30] benchmark database. For each of the alignments, a number of sequence features predicted by MACSIMS, such as domains or active site motifs, were compared to the known sequence features retrieved from the public databases. The specificity in these tests was shown to be above 99%, and the sensitivity was 91%. Two applications of MACSIMS are demonstrated. The first example addresses the problem of target identification in a high-throughput structural proteomics project, while the second example concerns the structural and functional characterisation of mutations known to be involved in human genetic diseases.

The total information content of the MACSIMS, including the mined data and the MACSIMS propagated or predicted information, is stored in an XML format file that is suitable for automatic, high-throughput projects. The source of all the information is also recorded in the XML file, so that an expert user can trace the origins of the new data. A web server is available for visual analyses, incorporating the JalView alignment editor [31,32]. JalView is a platform-independent program that is capable of displaying and editing large sequence sets, and allows multiple integrated views of the alignment and associated sequence features. This graphical presentation provides a valuable workbench for detailed functional analyses and integrated systems analysis.

## Implementation

### Construction of the test set

**Figure 1:**
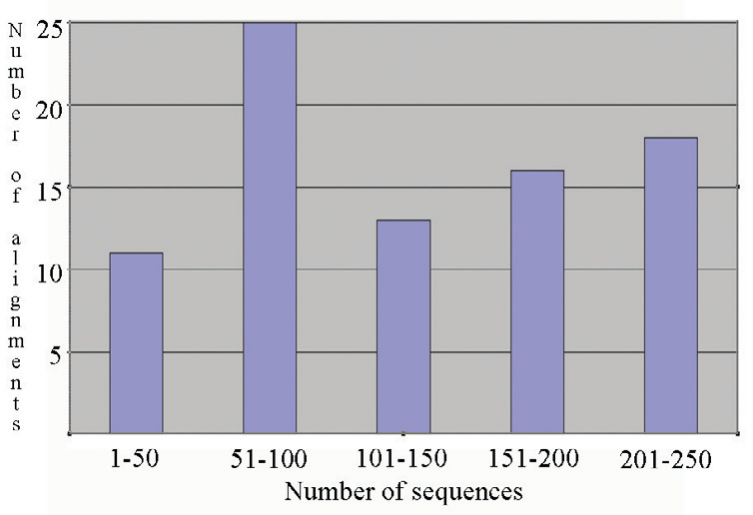
Distribution of the number of sequences/alignment in the well characterised data set used for validation.

A test set of multiple alignments was constructed based on the BAliBASE benchmark alignment database [30]. BaliBASE is designed for the evaluation and comparison of multiple sequence alignment algorithms and provides a large number of high quality, manually refined, reference alignments based on 3D structural superpositions. For each of the 83 multiple alignments in the reference set 1, a single PDB [27] sequence was selected from the sequences in the alignment, and the corresponding full-length sequence was extracted from the Swiss-Prot database [34]. All the sequences in a BAliBASE reference alignment share the same structural fold, so we selected the first sequence in the alignment as a representative sequence. For each of these initial query sequences, a BlastP search [35] was performed in the Uniprot and PDB databases. Next, a subset of sequences was selected from the sequences detected by BlastP with an Expect<10. The sample subset contained all the PDB sequences detected, together with a set of representative Uniprot sequences, built by dividing the sequences into 40 subgroups depending on the log of the BlastP expect values, and selecting one sequence from each subgroup. Sequences from the Swiss-Prot database were preferred because they are generally more extensively annotated than the SpTrembl sequences. Finally, the sequence subsets were aligned using the PipeAlign system [8]. The result is a test set of 83 automatically-constructed multiple alignments, containing a total of 10250 full-length sequences from the public sequence and structure databases. The distribution of the number of sequences per alignment is shown in Figure 1.

### Algorithm overview

**Figure 2:**
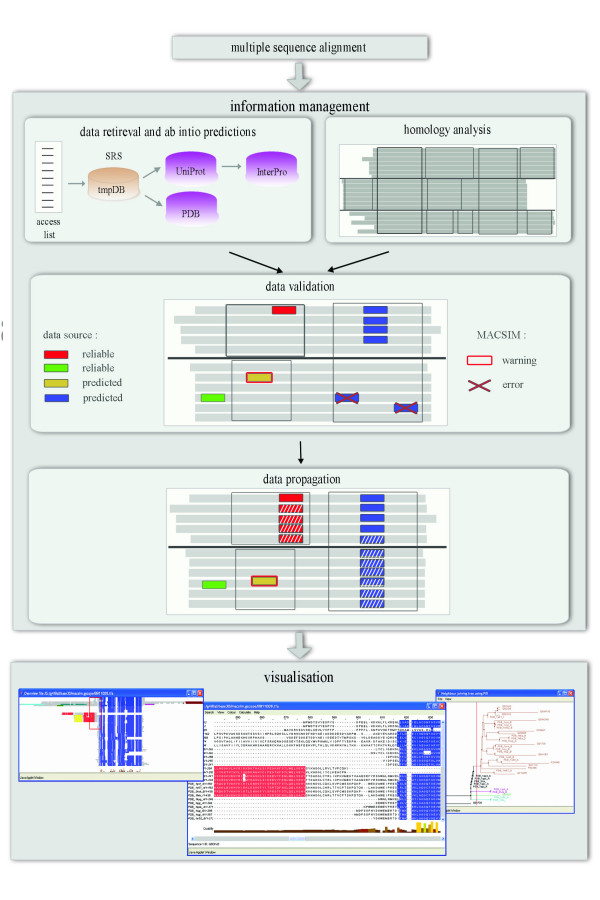
Schematic overview of the MACSIMS algorithm.

The algorithm incorporated in MACSIMS is composed of a number of integrated processes: (i) data retrieval and ab initio predictions, (ii) identification of homologous regions, (iii) data validation and propagation and (iv) data storage and presentation. The processing pipeline is outlined in Figure 2 and is described in detail below.

### Data retrieval and ab initio prediction

For each sequence in the multiple alignment, data is mined from the public sequence databases, using the SRS Sequence Retrieval System [28]. In order to retrieve information efficiently, a customized SRS database containing all the sequences in the alignment is created on-the-fly and this custom database is then indexed to the public database. If the sequence name in the alignment corresponds to a valid Uniprot [34] accession number, various sequence entry fields are retrieved first from Uniprot, including the organism name and NCBI TAXID, the function definition, GO definitions and the EC number, and the sequence feature table containing details of known domains, secondary structure elements (SSEs), functional or modified sites, etc. Pfam [36] domains and Prosite [37] patterns associated with each sequence are also retrieved via the InterPro database [38], although Prosite entries with a 'false' status are ignored. If the sequence name corresponds to a PDB [33] accession number, the function definition and the organism are retrieved from the TITLE and SOURCE fields respectively. The SSEs are also retrieved from the HELIX and SHEET fields. If the sequence name does not correspond to a valid database accession number, the sequence is kept in the alignment, although no data is mined. A number of different prediction programs are then run for each sequence: (i) the SEG algorithm [39] is used to identify low complexity segments, (ii) the GES hydrophobicity property [40] is used to predict potential transmembrane helices, (iii) coiled coil segments are predicted using the NCOILS program [41]. The information extracted from the sequence databases, together with the propagated and predicted information is stored in an XML format output file, based on the MAO multiple alignment ontology. The DTD for the MACSIMS XML format is available at .

### Identification of homologous regions

The reliable 'core blocks' in the multiple alignment are identified using the RASCAL algorithm [42], which combines a number of complementary sequence analysis algorithms in order to identify conserved, well aligned sequence segments. Briefly, the multiple alignment is first divided into sub-families using the Secator [43] sequence clustering program. Conserved blocks for each sub-family are then determined using the mean distance (MD) column scores implemented in the NorMD alignment objective function [44]. These subfamily core blocks are represented by profiles and are compared to each other in a pairwise fashion to identify sequence segments conserved between sub-families. Taking advantage of the transitive nature of homologous relationships, information from intermediate sequences is used to help define the conserved core blocks for more divergent sequences. Finally core blocks are chained into regions using the method developed in LEON [29]. These regions are characterized by their phylogenetic distribution, defined as the most specific taxon that is common to all sequences in the region.

### Data validation and propagation

The purpose of this step is to reliably transfer information from annotated sequences to unknown ones. However, in order to avoid propagating false information, the data has to be pre-processed to validate the data mined in step 1.

**Figure 3:**
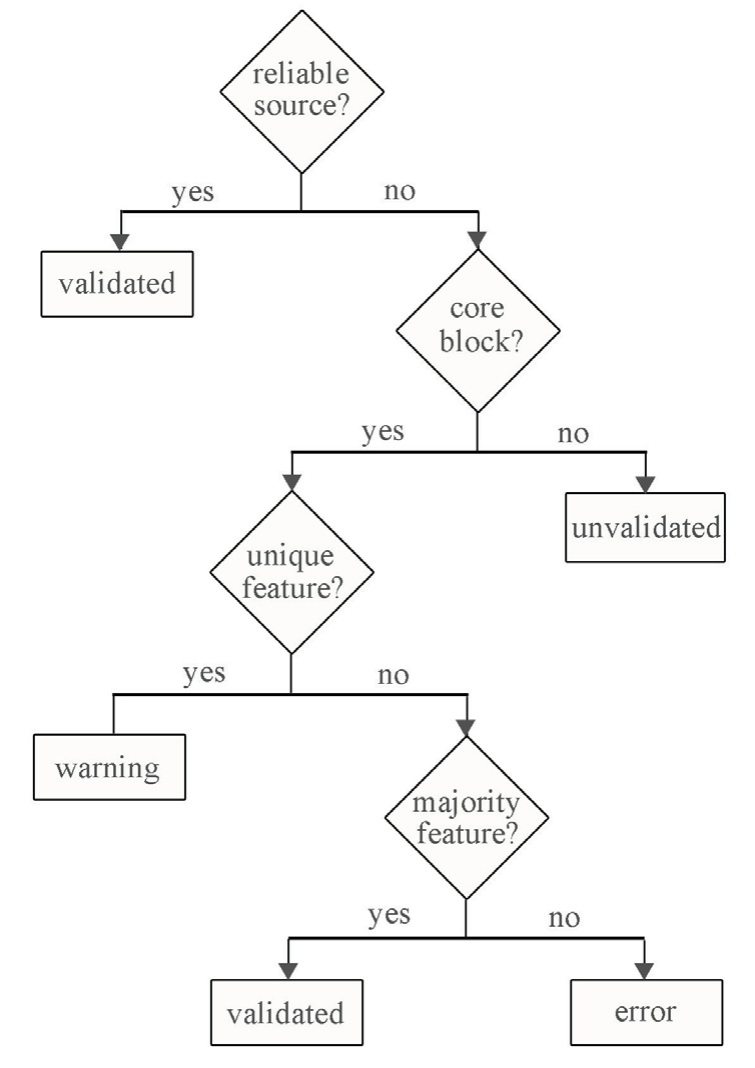
Decision rules for feature validation.

The mined data is first classified into a number of different sequence feature types, e.g. domain, SSE, active site or modified residue. (The full list of feature types is available on the MACSIMS web server help pages). A sequence feature is defined by its type, its start and end position within the sequence and an annotation text or 'name' that further characterises the feature. For example, the HMG_box domain of SSRP1_MOUSE [Swiss-Prot:Q08943] is defined as type = PFAM, start_residue = 547, end_residue = 615, name = 'PF00505 HMG_box'. We then use a series of decision rules to identify inaccurate or uncertain sequence features, as shown in Figure 3. Data from a 'reliable' source, such as Uniprot or PDB SSEs or PFAM domains is automatically assumed to be correct. Data from resources containing predicted information is validated only if it is located within a core block region and it is in agreement with the majority of the sequence features of this type (i.e. the sequence feature that has the maximal occurrence at this position in the alignment is assumed to be the correct one). Unique predictions within a core block region are labelled with a 'warning' and are not included in the propagation step described below.

**Figure 4:**
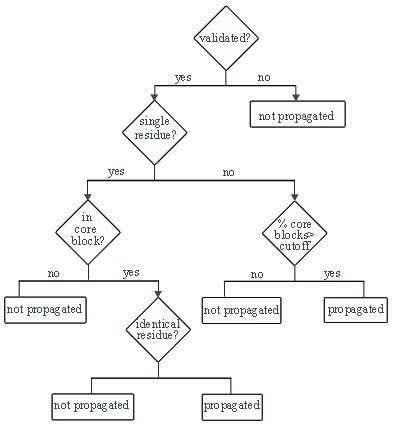
Decision rules for feature propagation.

Once the unreliable features have been identified, the remaining features are propagated between the sequences in the alignment, depending on a number of pre-defined criteria as shown in Figure 4 and Table 1. Thus, a feature is propagated from an annotated sequence to an unknown target sequence, if the following conditions are met. (i) For single residue sites, the site must be located within a core block that is present in both the annotated and the target sequence. Also, the residue in the annotated sequence corresponding to the site and the aligned residue in the target sequence must be identical. (ii) For SSEs, at least 70% of the element must be covered by core blocks shared by the annotated and the target sequence. (iii) For domains, at least 40% of the domain must be covered by core blocks shared by the annotated and the target sequence. The threshold values used for these conditions were determined by manual inspection of the MACSIMS results using the original BAliBASE reference alignments. The values were chosen to maximize the specificity of the MACSIMS propagation i.e. a cutoff was selected that removed all false positive predictions in this initial training set. Sequence features that do not satisfy these conditions are stored and presented to the user, but are not propagated to any other sequences.

### Data storage and presentation

The final output after the propagation step is an XML format file containing the complete MACSIMS knowledge base. The sequence features that are generated by MACSIMS are annotated as either Predicted or Propagated features. In the case of propagated features, the source sequence name is included in the annotation. The XML format provides an appropriate format for automatic parsing of the results that facilitates integration in high-throughput systems. The XML format also provides the possibility to include a reliability score for each sequence feature. These scores will be improved in future versions of MACSIMS in order to allow expert users to further validate borderline features using other tools. Files are also generated for input to the JalView applet (see Figure 2). JalView is a Java application for editing and viewing sequence alignments. It has facilities for assessing alignment quality, visualizing residue property conservation, and constructing and viewing sequence clusters through tree and principal components based algorithms.

## Results

MACSIMS is a new information management system that combines data from a number of different resources with computational methods for data validation and analysis. The structural and functional information retrieved from various public databases is first validated in the context of the MACS. The validated information is then used to characterise the unknown proteins. Thus, MACSIMS provides detailed annotations ranging from the location of active sites and the definition of structural or functional domains to the description of the protein function at the molecular or cellular levels. In an initial large-scale test; a set of well characterised proteins is used to evaluate the specificity and sensitivity of MACSIMS. Then, in a second test, MACSIMS is used to structurally and functionally characterise mutations known to be involved in human genetic diseases. The results of the analyses are represented in a format designed specifically for high-throughput computational processing. The results can also be examined manually by the biologist using a user-friendly, web-based interface.

### Benchmarking with a large test set of well characterised protein families

In order to evaluate the quality of the data management algorithms incorporated in MACSIMS, we used a large-scale test set based on the BAliBASE benchmark alignment database. Version 3 of BAliBASE contains representative test cases that cover most of the protein fold space, divided into 5 reference sets representing many of the problems encountered when aligning real families of proteins. The protein families in BAliBASE are well characterised and the known information can be used to validate the predictions made by MACSIMS. However, the reference alignments in this database are based on 3D structure superpositions and have been manually refined to correct any misalignments. In order to provide realistic test cases for MACSIMS, we therefore selected an initial set of 83 query sequences that were used to construct automatic multiple alignments containing full-length sequences (see Methods). These test alignments are thus representative of typical results that would be obtained in an automatic protein annotation pipeline. Information was collected from the public databases for all sequences in each alignment. The 83 alignments contained a total of 10250 sequences, with 8045 PDB sequences and 2205 Uniprot sequences. After the data retrieval step, the MACSIMS alignments contained 9799 functional definitions and 4069 GO (Gene Ontology) crossreferences. Taxonomic data included 8714 organism names and 17322 taxon entries. A total of 150772 sequence features were retrieved from the databases, including 8020 Interpro entries and 121858 secondary structure elements (SSEs). The consensus prediction algorithms contributed a further 2535 predicted features (903 low complexity segments, 937 transmembrane helices, 695 coiled coils). During the data validation process, 84 of the mined features were identified as potential errors and were excluded from the propagation step. An additional 261791 sequence features were generated by propagation of the validated features. The *ab initio *prediction algorithms used in MACSIMS are standard methods that have been described elsewhere. Therefore, the results we present here concern only the feature validation and propagation steps.

The sensitivity and specificity of this process were measured in a test designed to illustrate the behavior of MACSIMS for different kinds of sequence features, i.e Pfam domains, Prosite motifs, SSEs and functional sites from the Uniprot feature table. The protocol used in the test is as follows:

1. For all 83 test alignments, the feature retrieval and validation steps were repeated as described above, resulting in 150772 sequence features.

2. The retrieved sequence features were removed from the query sequence and any other sequences sharing >90% residue identity with the query. A total of 15697 features were removed, of which 2283 belonged to the query sequence. This leaves a total of 135075 sequence features in the remaining sequences (sharing <90% identity with the query) in the alignment.

3. Finally, the propagation step was performed as before.

The features propagated by MACSIMS to the query sequences were then evaluated by comparison to the 2283 excluded query features. A propagated feature that overlapped an excluded feature with the same name was considered as a true positive (TP), while a propagated feature that overlapped an excluded feature with a different name was considered as a false positive (FP). Only one FP feature was observed: for the sequence [Swiss-Prot:P01843] (corresponding to [PDB:1JNH_A]), the Uniprot feature table indicates a strand at position 9–29, whereas MACSIMS has propagated a strand-helix-strand configuration in this segment. The MACSIMS propagation is in fact in agreement with the annotation of 1JNH_A in the PDB database, indicating a potential error in the Uniprot feature table entry. The specificity (FP rate) is difficult to estimate accurately as the total number of true features for the query sequences is unknown. The public databases contained 2283 features related to the query sequences, but this does not necessarily represent the complete set of possible features. If we consider only the propagated features that contradict known information, only one FP was detected as described above and the specificity is estimated to be over 99%. MACSIMS correctly predicted 76% of the 2283 original query sequence features. Two main reasons for this relatively low sensitivity (TP rate) were identified. First, some of the excluded features did not exist in any of the homologous sequences in the multiple alignment. Second, local alignment errors occurred that resulted in misaligned sequence features. As no core blocks were defined for these segments, the sequence features could not be propagated. If these sequence features are omitted from the analysis, the sensitivity of MACSIMS for the propagation of correctly aligned sequence features is 91%.

The same protocol was repeated in another test, where the same features were removed from sequences sharing more than 50% identity with the query. Even for this difficult test case, in which only distantly related sequences are used in the feature propagation step, the specificity of MACSIMS remains >99% with only one propagated feature in disagreement with the known annotations. The sensitivity of MACSIMS is only slightly lower, with 86% of the features correctly aligned in the multiple alignment being successfully propagated. Details for the different kinds of features are shown in Table 2. For each type, the number of known features associated with the query sequences is indicated. The complete set of features retrieved for the other sequences in the alignment is used as a basis for propagation. The number of features in this complete set that correspond to known query features is indicated in the table as 'homolog features'. For example, the 83 queries contained 360 known functional sites in the UniProt database, but only 305 of these features was also found in one of the other sequences in the alignment. The known features are then compared to the features propagated by MACSIMS and the number of true positives and false positives are calculated. For the Pfam domains and the Prosite motifs which are available for all the sequences in the Uniprot database, most of the known query features are successfully recovered by MACSIMS. In contrast, the propagation of SSEs and Uniprot functional sites, which are available only for experimentally validated sequences, is less effective.

In these tests, MACSIMS predicted a total of 1060 new features for the query sequences that did not overlap any of the excluded features. Some of these propagations were verified manually by reference to text annotations or literature references. For example, the active site of the query protein THIO2_ANASP [Swiss-Prot:P20857] was propagated from sequence P80028, which shares 24% residue identity. The site has been described as essential for the catalysis of redox reactions [45]. As a second example, for the query sequence LDH1_PLAFD [Swiss-Prot:Q27743], the L-lactate dehydrogenase active site (PS00064) was identified as a new feature. This site is described as a known false negative in the Prosite database. The MACSIMS alignments for all the tests are available for viewing on the web at .

### Large scale application of MACSIMS

MACSIMS has been integrated in a high-throughput structural proteomics project (SPINE), where it was used for target selection and characterization [46]. It is also being used in the MS2PH (Structural Mutation to Human Pathologies Phenotype) project, in order to analyse proteins involved in human genetic diseases and to identify mutations that cause structural or functional perturbations. This application illustrates the data integration potential of MACSIMS by characterizing mutations in terms of their evolutionary conservation, their position in the 3D structure, and their role in functional sites. For an initial test set of 100 proteins with well characterized mutations, a BlastP search was performed in the Uniprot and PDB databases and a MACS was constructed of the 100 top scoring sequences using the PipeAlign system. For the 100 query proteins, a total of 2377 sequence features were mined directly from the public databases. MACSIMS propagated an additional 1424 features from homologous sequences in the MACS, representing a 60% increase in the number of features identified. A further 300 features, including low complexity segments, coiled coil segment and transmembrane helices were predicted by *ab initio *methods. The initial data set of 100 proteins and the MACSIMS alignments for all these tests are available for viewing on the web at .

**Figure 5:**
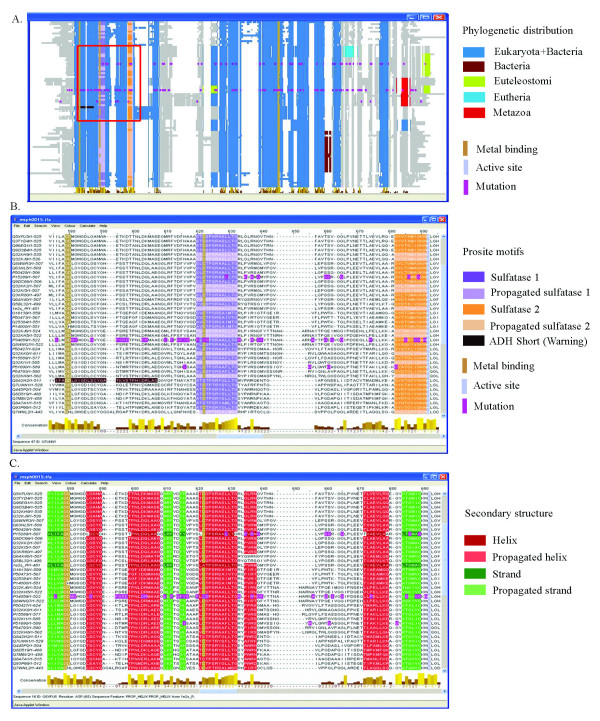
Example MACSIMS alignment analysis presented in the JalView applet. A. Overview of complete alignment. Regions calculated by MACSIMS are coloured according to their phylogenetic distribution. The red box indicates the section of the alignment shown in B and C. A conservation score [50] for each alignment column is shown below the alignment. B. Detailed view of one part of the alignment. Metal binding and active site residues are indicated, together with Prosite motifs. Mutated residues are shown with a pink background. C. The same part of the alignment as in B, with secondary structure elements highlighted.

An example MS2PH alignment of the sulfatase protein family is shown in Figure 5. Figure 5A shows a schematic overview of the complete alignment produced by JalView, in which the regions calculated by MACSIMS are coloured according to their phylogenetic distribution. The metazoa-specific region identified in the alignment corresponds to a 7 kDa chain (component C) of the arylsulfatase A precursor, which is linked to component B by disulfide bonds. The positions of known point mutations in three human sequences are also indicated: arylsulfatase E precursor [Swiss-Prot:P51690], N-acetylgalactosamine-6-sulfatase precursor (P34059) and arylsulfatase A precursor [Swiss-Prot:P15289]. Figure 5B shows a detailed view of the alignment of the N-terminal metal-binding domain, highlighting the mutations in this zone and the relative positions of the predicted Prosite motifs. The two motifs, sulfatase 1 and 2 are consistently predicted in a number of different sequences and are propagated to the unannotated sequences in the alignment. However, the ADH short motif is not propagated by MACSIMS because it is only present in one sequence and may correspond to a false positive prediction in the Prosite database. Figure 5C corresponds to the same alignment zone, showing the position of the mutations in relation to SSEs and important functional sites. For example, the C79Y mutation in the GALNS gene (alignment column 622), which occurs at the catalytic site, leads to serious damage in GALNS activity and a severe phenotype [47].

## Discussion

MACSIMS can be used to integrate information from different domains, such as genetics, structural biology, proteomics or interactomics experiments, in the context of a multiple alignment of a protein family. Input alignments can be obtained by any of the new automatic programs, such as PipeAlign [14], MAFFT [13], MUSCLE [15] or ProbCons [16], or manually constructed. For those sequences in the alignment with Swissprot or TrEMBL accession numbers, information is automatically mined from public databases. The major advantage of MACSIMS is that the mined information can be cross-validated within the alignment, in order to differentiate between reliable, consistent information and spurious predictions. The validated data then provides the basis for the accurate propagation of information from known to unknown sequences.

An important factor in the design of MACSIMS was its potential application in automatic, high-throughput systems. It was therefore crucial that the data propagation system should be reliable and should clearly identify the source of all inferences. The data collected by MACSIMS will inevitably contain a number of errors, either from high throughput experimental techniques or from computational prediction methods. These errors in the input data can be handled in two different ways, either by leaving the noisy instances in and using a robust algorithm that is not biased by the noise, or by filtering the data before use. In the second approach, instances that are suspected of being noisy according to certain evaluation criteria are discarded. We chose the second approach because our datasets are generally not large enough to permit robust statistical inferences. A non-parametric decision tree was implemented that identifies and removes suspicious predictions, resulting in a smaller but cleaner data set for propagation.

Another important issue is the level of similarity required in order to transfer information between different proteins. The automatic annotation of proteins based on the transfer of function descriptions from the most closely related homolog has lead to a number of errors in high-throughput genome annotation projects. Some common causes of questionable predictions are: i) non-critical use of annotations from existing database entries; ii) taking into account only the annotation of the closest homolog; iii) insufficient masking of low complexity segments in protein sequences, resulting in spurious database hits iv) ignoring multi-domain organization of the query or target proteins [48]. In MACSIMS, we have addressed this problem by applying a recently developed method to identify the well-aligned, homologous regions in the alignment. Information is then propagated only within these safe regions. As a corollary, the sensitivity of the MACSIMS propagation algorithm will depend on the quality of the input alignment. If core blocks are not identified due to errors in the alignment, no propagation can occur. Although significant progress has been made recently in the quality of automatic multiple alignments, errors do still occur when aligning large sets of complex proteins. Nevertheless, in the large scale tests using automatically constructed alignments, we have shown that the sequence annotations are significantly increased compared to the available database information, leading to a more complete knowledge base for subsequent analyses. Even in the extreme case of protein families with no known homologs in the public annotated databases, MACSIMS can provide useful information, such as the clustering of the sequences into sub-families and the identification of conserved regions within sub-families or in the full alignment. Also, the predictions of transmembrane regions, coiled coil segments and low complexity regions may give some clues as to the potential role of the protein family.

The SRS system is currently used in the data retrieval step as it provides a single interface for most general biological databases, and allows a fast access because of on-the-fly database for sequences in alignment. Information is retrieved from the Uniprot sequence and PDB 3D structure databases. Uniprot provides links to domain information, including the Interpro database of protein families, domains and functional sites, as well as to experimental information related to structure, function, mutations and disease. In the future, other data resources will be incorporated, such as the NCBI sequence resources and the interaction and mutation databases. An alternative data retrieval system will be implemented that will include a remote access facility to these new resources. Another abundant data source that could be exploited is the scientific literature, thanks to the development of new methods and tools for literature-mining [49].

The MACSIMS system is currently available as an interactive web server. A web service using the SOAP  protocol is planned for the near future. All the information collected or generated by MACSIMS is stored in XML format files that provide a structured format for automatic data parsing by computers. However, all the information is also easily accessible for manual analysis by biologists, via new enhancements to the JalView editor. JalView provides a simple-to-use, graphical interface suitable for non expert users that offers an interactive environment for in-depth protein family analysis.

## Conclusion

MACSIMS is a new system for the management of all the information related to a protein family. Structural and functional information is automatically retrieved from the public databases. The advantage of MACSIMS is that the raw data can be validated in the context of the multiple alignment and information can be propagated from known to unknown proteins. MACSIMS thus provides a unique environment that facilitates knowledge extraction and the presentation of the most pertinent information to the biologist.

Work is now in progress to incorporate MACSIMS in the PipeAlign protein family analysis WWW server. The new version of this server will allow automatic processing of proteins, from database searches for homologous sequences and construction of a high-quality, validated MACS to information and management using MACSIMS. MACSIMS will also be integrated in a new system, Ordalie (Ordered Alignment Information Explorer) that will allow detailed residue conservation analysis at the complete family or the sub-family level, in order to characterize sub-family specific residues and differentially conserved motifs. We are also investigating the application of recent developments in ontology-based methods for reasoning and inference that will facilitate intelligent knowledge extraction and decision support for structural, functional or evolutionary analyses.

## Availability and requirements

MACSIMS consists of a suite of programs, all written in ANSI C. The programs were installed and tested on a DEC Alpha 6100 computer running OSF Unix. MACSIMS uses the SRS system (version 7.1.3.2) and the Uniprot, PDB and Interpro databases, which are updated weekly. A Web server is available at  that runs the complete suite of programs for a given multiple alignment. A UNIX shell script is also provided for users wishing to run the system locally. In this case, the SRS system must be installed for data retrieval. SRS runs on Unix/Linux systems and requires at least 100 Mb of RAM plus enough disk space to hold the databases. The Secator program  is also needed for sequence clustering and the NCOILS program  is required for the prediction of coiled coil segments. MACSIMS takes multiple alignments in any of the most widely used formats, including MSF, FASTA or ClustalW formats as input. For successful data retrieval, the sequence names should correspond to Uniprot accession numbers and the sequences should be full length. MACSIMS outputs an alignment in XML format, that can also be converted into the input format required by the JalView program.

## Authors' contributions

JDT and OP both contributed to the design of MACSIMS. JDT developed the main methodology and drafted the manuscript. AM and FP developed the data retrieval methods and FP implemented the web server. AW, JP and GJB developed and enhanced the JalView alignment display applet. OP supervised and coordinated the project. All authors read and approved the final manuscript.

## Figures and Tables

**Table 1:** feature propagation criteria

Feature type	Description	Data source	Feature category	Core block Coverage
DOMAIN	Structural/functional domain	Uniprot (predicted)	Domain	40%
PFAM-A	Pfam database domain	Pfam (reliable)	Domain	40%
PROSITE	Prosite motif or domain pattern	Prosite (predicted)	Single residue Domain	100% 40%
STRUCT	Secondary structure element	Uniprot/PDB (reliable)	SSE	70%
MODRES	Modified residue	Uniprot (predicted)	Single residue >1 residue	100% 70%
SITE	Active site	Uniprot (predicted)	Single residue >1 residue	100% 70%
VARSPLIC	Splicing variant	Uniprot (not propagated)	N/A	N/A
VARIANT	Residue variants or mutations	Uniprot (not propagated)	N/A	N/A
BLOCK	Conserved core block	Calculated in MACSIMS	N/A	N/A
REGION	Conserved region	Calculated in MACSIMS	N/A	N/A
LOWCOMP	Low complexity segment	Calculated in MACSIMS	N/A	N/A
TRANSMEM	Potential transmembrane helix	Calculated in MACSIMS	N/A	N/A
COIL	Potential coiled coil	Calculated in MACSIMS	N/A	N/A

The data source indicates the original database from which the feature type is retrieved (predicted indicates a feature type that may contain predicted/unreliable information; reliable indicates a feature type that is assumed to manually verified/reliable). The Feature category refers to the three categories used to determine the criteria for feature propagation. Core block coverage indicates the percentage of the feature that should be covered by core blocks for the feature to be propagated.

**Table 2:** benchmark test results

Feature type	percent identity	query features	homolog features	true positive	false positive	new features
PFAM-A domain	<90%	161	161	160	0	4
	<50%	161	150	149	0	3
PROSITE pattern	<90%	166	165	160	0	5
	<50%	166	153	148	0	4
Uniprot site	<90%	360	305	260	0	64
	<50%	360	288	235	0	56
secondary structure	<90%	1486	1265	1150	1	987
	<50%	1486	1197	1009	1	802

Total	<90%	2283	1896	1730	1	1060
	<50%	2283	1788	1541	1	865

Percent identity indicates the maximum similarity of the sequences in the alignment with the query. 'Query features' is the number of sequence features for the query available in the public databases. 'Homolog features' is the number of features found in the other sequences in the alignment that correspond to a feature in the query. True (or false) positives indicate the number of features propagated by MACSIMS that match (or mismatch) with known query features. 'New features' is the number of features predicted by MACSIMS that are not currently found in the public databases.
